# A novel missense mutation in the *MECOM* gene in a Chinese boy with radioulnar synostosis with amegakaryocytic thrombocytopenia

**DOI:** 10.1186/s12887-024-04552-1

**Published:** 2024-01-20

**Authors:** Duowen Huang, Mingyan Jiang, Yiping Zhu, Dongjun Li, Xiaoxi Lu, Ju Gao

**Affiliations:** 1 Department of Pediatric Hematology and Oncology, West China Second University Hospital, Sichuan University, No. 20 Section 3, South Renmin Road, Chengdu, 610041 Sichuan Province China; 2 Key Laboratory of Birth Defects and Related Diseases of Women and Children (Sichuan University), Ministry of Education, Chengdu, 610041 Sichuan Province China

**Keywords:** Radioulnar synostosis with amegakaryocytic thrombocytopenia (RUSAT), *MDS1 and EVI1 complex locus (MECOM)* gene mutations, Whole-exome sequencing (WES), Hematopoetic stem cell transplantation

## Abstract

Radioulnar synostosis with amegakaryocytic thrombocytopenia (RUSAT) type 2, caused by *MDS1 and EVI1 complex locus* (*MECOM*) gene mutations, is a rare inherited bone marrow failure syndrome (IBMFS) with skeletal anomalies, characterized by varying presentation of congenital thrombocytopenia (progressing to pancytopenia), bilateral proximal radioulnar synostosis, and other skeletal abnormalities. Due to limited knowledge and heterogenous manifestations, clinical diagnosis of the disease is challenging. Here we reported a novel *MECOM* mutation in a Chinese boy with typical clinical features for RUSAT-2. Trio-based whole exome sequencing of buccal swab revealed a novel heterozygous missense mutation in exon 11 of the *MECOM* gene (chr3:168818673; NM_001105078.3:c.2285G > A). The results strongly suggest that the variant was a germline mutation and disease-causing mutation. The patient received matched unrelated donor hematopoetic stem cell transplantation (HSCT). This finding was not only expanded the pathogenic mutation spectrum of *MECOM* gene, but also provided key information for clinical diagnosis and treatment of RUSAT-2.

**Supplementary Information:**

The online version contains supplementary material available at 10.1186/s12887-024-04552-1.

## Introduction

Radioulnar synostosis with amegakaryocytic thrombocytopenia (RUSAT [MIM: 605,432]), first described by Nielsen and colleagues in 2012 [1], is a rare inherited bone marrow failure syndrome (IBMFS) with a continuous spectrum of clinical phenotype from isolated proximal radioulnar synostosis to severe bone marrow failure without skeletal abnormalities in the patients and affected relatives [2]. Some of the patients were complicated with renal and cardiac malformations, and hearing loss [3, 4].

So far, about 64 variants have been reported, with 5 reported Chinese individuals and family members in the literature reviewed by Voit RA, et al. [5]. Type 2 of RUSAT (RUSAT-2; MIM: 616,738) is caused by a mutation in the *MECOM* gene (MIM: 165,215) on chromosome 3q26. *MECOM* is an abbreviation of *MDS1* (myelodysplasia syndrome 1) and *EVI1* (ecotropic viral integration 1 site) *complex locus*, which encodes a 1051 amino acid protein and contains 10 zinc finger motifs and its isoforms act as transcriptional factors [2, 6, 7]. RUSAT-2 is not an easily recognizable syndrome due to the limited knowledge of reported cases and the heterozygous clinical manifestations, especially in the cases without bone abnormalities. To our knowledge, all reported RUSAT-2 cases were diagnosed through next generation sequencing and most of them received HSCT.

In the present study, we describe a 1-year-3-month-old Chinese boy with a novel heterozygous missense variant in the *MECOM* gene identified by next generation sequencing and treated by HSCT. In addition, the genetic and phenotypic spectrum and the outcome of reported RUSAT-2 cases are reviewed and summarized.

## Case report

**Fig. 1:**
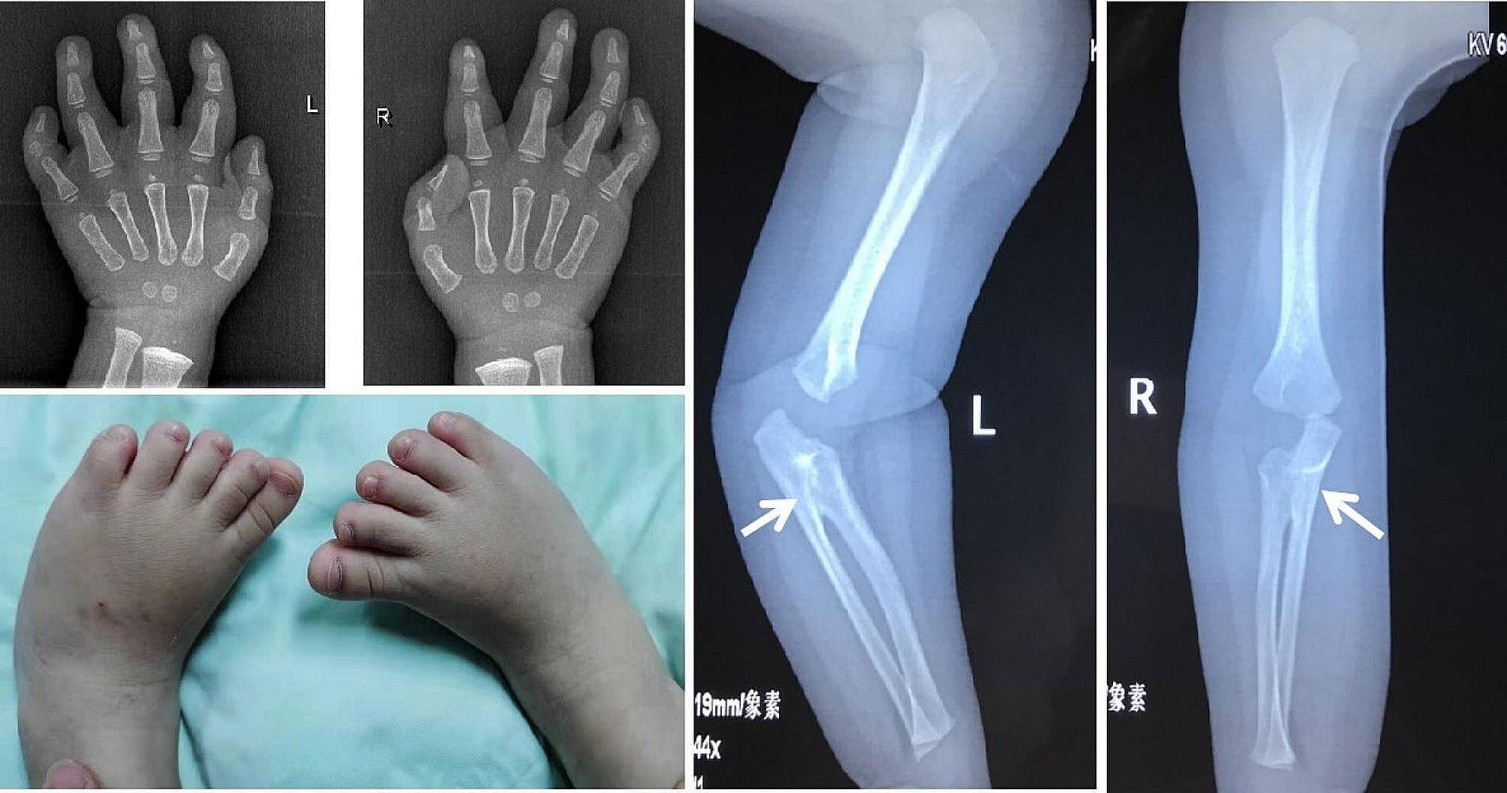
Radiographs of the patient show bilateral radioulnar synostosis (white arrows). Bilateral hands and feet were clinodactyly of digits

The reported patient was a 1-year-3-month-old Chinese boy. He was referred to our hospital due to petechiae, pallor, and refractory fever for one year. He was the first born of a non-consanguineous Chinese couple, and there was no family history of hematological disorders. The pregnancy and delivery at 39 wk gestation were uneventful. At birth, the weight was 3200 g, the length was 49 cm, and the occipitofrontal circumference (OFC) was 32.5 cm. His hearing and psychomotor development were normal. He did not have facial dysmorphism, microcephaly or organomegaly, but had bilateral clinodactyly of the digits without abnormalities of bones. Bilateral forearms were limited to abduction and internal rotation (Fig. 1).

For the proband, regular laboratory tests were performed. Complete blood count revealed pancytopenia (minimum value of neutrophil count was 0.07 × 10^9^/L (normal reference range 0.8 × 10^9^/L-5.8 × 10^9^/L), minimum value of hemoglobin was 17 g/L (normal reference range 110 g/L-160 g/L), minimum value of platelet count was 1 × 10^9^/L (normal reference range 100 × 10^9^/L-300 × 10^9^/L), reticulocyte count of 1.9%). His liver and kidney function, electrolytes, myocardial enzymes, thyroid function, erythrocyte sedimentation rate (ESR), hemoglobin electrophoresis, and coagulation profile were normal. The virus infectious screening, Coombs’ test, and autoimmune antibody were negative. Bone marrow smear revealed hypocellular, megacaryophthisis. Chromosomal breakage study was normal. Single cell gel electrophoresis (SCGE) revealed damage of lymphocytes in peripheral blood, and the rate of cometal cells was 22% (normal range < 21%). His CD19 positive B-cell count was 0.09 × 10^9^/L (normal range 0.20–2.10 × 10^9^/L). The karyotype was 46, XY.

The X-ray of both forearms showed superior radioulnar synostosis. The X-ray of both hands and feet showed clinodactyly and brachydactyly (Fig. 1). Computerized tomography (CT) of brain revealed subarachnoid hemorrhage. There was no abnormality in his CT of chest and abdomen, echocardiography, ultrasonic test of urinary system, or hearing screening.

**Fig. 2:**
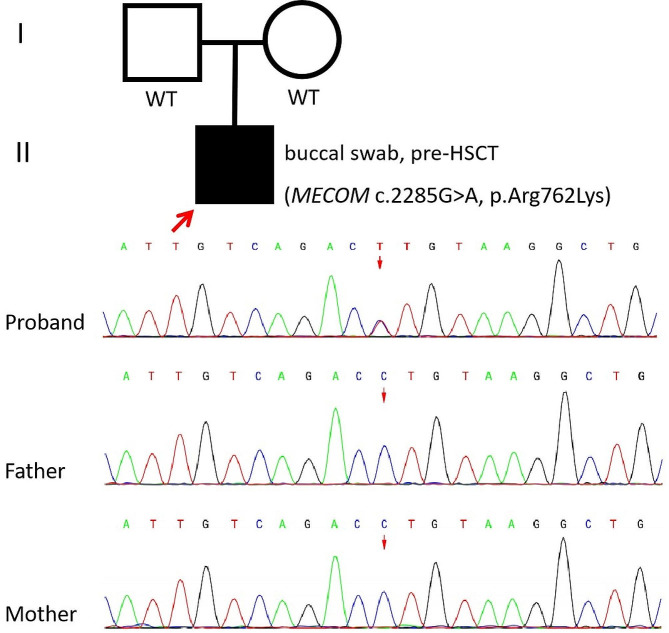
Family pedigree (the proband is marked with big red arrow) and Sanger sequencing diagram of the *MECOM* variant (c.2285G > A, p.Arg762Lys). The red arrows indicate the substitution present in the patient, but absent from his parents. WT, wild-type

We performed trio-based whole-exome sequencing (WES) on the buccal swab and peripheral blood of the patient and peripheral blood of his unaffected parents. Genomic DNA was extracted from buccal swab and peripheral blood. The captured DNA fragments were then sequenced at the Chigene Translational Medical Research Center (Beijing, China). The sequence variants were functionally annotated and filtered using known populations and databases, including 1000 genomes, Signle Nucleotide Polymorphism Database, Genome Aggregation Database, Clin Var, Human Gene Mutation Database, and Online Menedelian Inheritance in Man. Candidate variants were then evaluated in the context of clinical presentation and inheritance mode. The trio-based WES revealed a heterozygous missense variant c.2285G > A in exon 11 of the *MECOM* gene (NM_001105078.3) in the proband, predicted to result in the amino acid substitution of arginine for lysine at codon 762 (p.Arg762Lys). The variation was not reported previously. No variation was detected at this site in his parents, which was subsequently validated by Sanger sequencing (Fig. 2). The *de novo* variant was not observed in public variant databases. This novel *de novo* variant was classified as “likely pathogenic” according to the American College of Medical Genetics and Genomics (ACMG) standards and guidelines [8].

He was diagnosed as RUSAT-2, and treated with intravenous immunoglobulin, methylprednisolone, antibiotics, platelet and packed red cells products. The petechiae and intracranial bleeding disappeared, but he still had pancytopenia. Follow up at 1 year revealed persisting anemia with severe neutropenia and thrombocytopenia. He had been treated with androgen for one month and intermittently supported with blood products. But he was dependent on transfusion, so he received matched unrelated donor hematopoetic stem cell transplantation (HSCT) at the age of 1 year 4 months. After transplantation, the rate of transfusion was decreased with less frequent infection. The last follow-up was at 4 years old. The complete blood count after HSCT was normal (neutrophil count 3.82 × 10^9^/L, hemoglobin 122 g/L, platelet was 317 × 10^9^/L).

## Discussion

RUSAT-2 is a rare autosomal dominant bone marrow failure syndrome (IBMFS) caused by mutations in the *MECOM* gene. As reported, the clinical manifestations of the cases with *MECOM* mutations presented a continuous spectrum from isolated radioulnar synostosis to severe bone marrow failure without skeletal abnormalities, which are named as *MECOM-*associated syndromes [2, 9]. Radioulnar synostosis was observed in 45.3% of patients with *MECOM* mutations reported as RUSAT-2. The other relatively prevalent features, as observed in 42.2% of reported RUSAT-2 cases, were other skeletal malformation, including hypoplasia of middle and end phalanx D5, Toe malposition D2, Thumb under D2. Pancytopenia was the dominant hematologic abnormality (36/64, 56.2%), though 8/64 (12.5%) patients had no cytopenia, and 16/64 (25.0%) patients had thrombocytopenia. Additionally, other malformations such as cardiac malformations (27/64, 26.6%), hearing impairment (9/64, 14.1%), nail, or facial abnormalities (15/64, 23.4%), renal (6/64, 9.4%) and neurological disorder (11/64, 17.2%), and precocious puberty (4/64, 6.3%) have been reported in patients with *MECOM* mutations, but were not seen in our patient (Supplementary Table 1) [1–4, 7, 9–23]. Notably, our patient had consulted an orthopedist for bilateral clinodactyly of the digits. It has been implied that some patients with RUSAT-2 may be initially misdiagnosed as isolated orthopedic disorders. Although the CT brain revealed subarachnoid hemorrhage, the patient had no neurological abnormalities. We speculated that the hemorrhage was caused by severe thrombocytopenia. Interestingly, immune dysfunction, such as decreased B cell and hypogammaglobulinemia had been observed in some patients (10/64, 15.6%) (Supplementary Table 1) [1–4, 7, 9–23]. It seems that *MECOM* is involved in critical pathways for regulation of regenerative hematopoiesis and B-cell differentiation. RUS (radioulnar synostosis) and B-cell lymphopenia has been observed only in patients with mutations affecting a short region in the C-terminal zinc finger domain of *EVI1* [2]. But it is not clear whether B-cell deficiency in *MECOM*-associated disease is due to a common stem-cell defect or to the specific involvement of *MECOM* in B-cell development. A specific role in B-cell development is suggested by the fact that a gene amplification of *MECOM* seems to play a role in persistent polyclonal binucleated B-cell lymphocytosis [24].

Following a thorough review of all reported RUSAT-2 cases, we considered the remarkable features distinct from RUSAT-2 were proximal radioulnar synostosis and thrombocytopenia. However, because the thrombocytopenia is nonspecific and skeletal abnormalities are insidious, and the total number of reported cases is limited, WES technology is increasingly used to identify the pathogenesis and establish a definite diagnosis. We performed trio-based WES and identified a novel *de novo* missense mutation (NM_001105078.3:c.2285G > A, p.Arg762Lys) of the *MECOM* gene in our patient. The variant was classified as “likely pathogenic” according to the ACMG criteria, supporting a genetic diagnosis of RUSAT-2 for the proband, with main complaints of pancytopenia and forearms limited movement.

Together with the reported cases, 64 different *MECOM* variants in 64 unrelated individuals and family members with RUSAT-2 were known. Of the detected pathogenic or likely pathogenic mutations, 6 (9.4%) cases were nonsense mutations, 5 (7.8%) cases were splice mutations, 8 (12.5%) cases were deletion, 6 (9.4%) cases were frameshift mutations, and 35 (54.7%) cases were missense mutations, while 4 patients were unknown the mutation types [1–4, 7, 9–23]. The majority of the reported mutations were missense mutations. The region coding for the 10 zinc finger domains are the mutational hotspots. The case described here presented a variant in exon 11 leading to an amino acid exchange at the 9^th^ zinc finger.

**Fig. 3:**
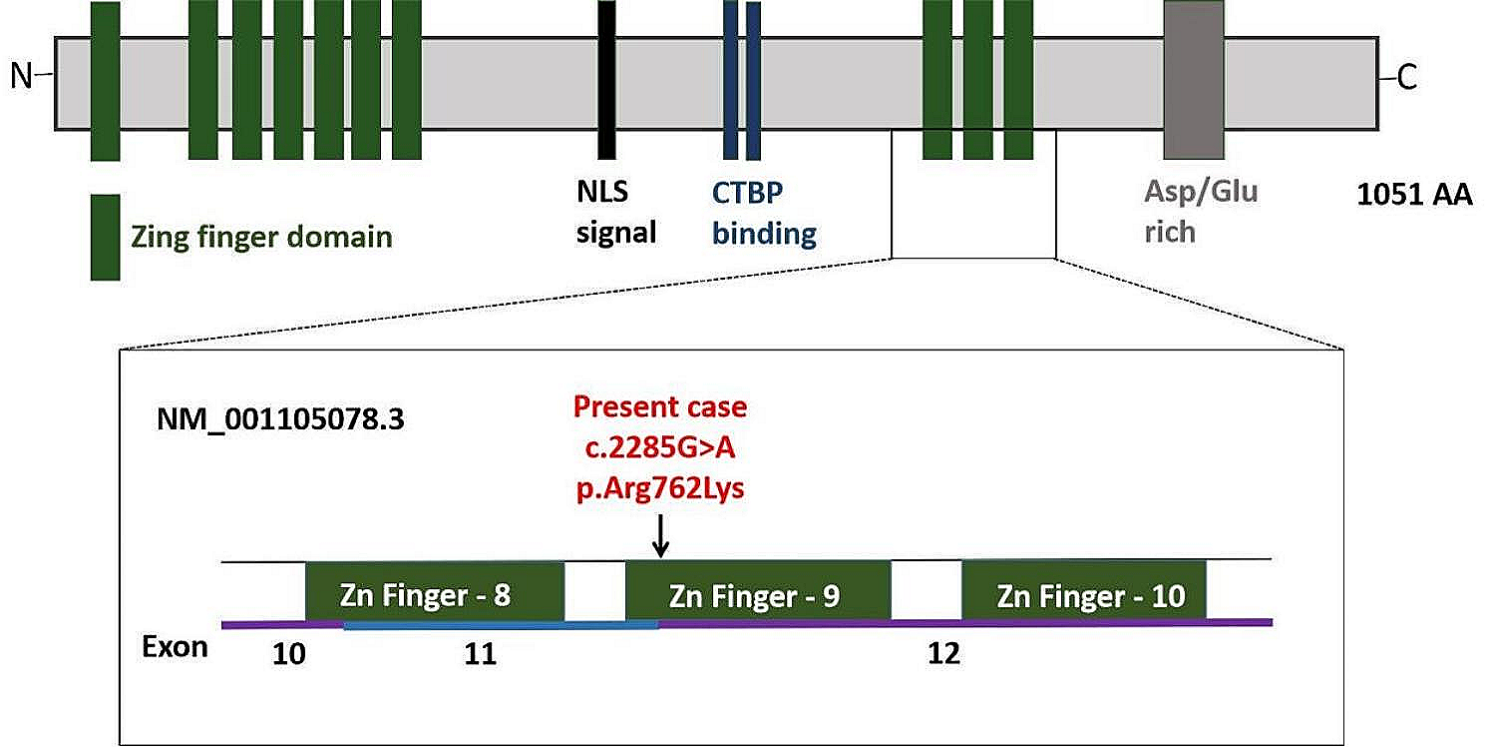
*MECOM*: MDS1 and EVI1 complex locus on Chr 3q26.2. *EVI1* gene and isoform were indicated. Location of patient variant indicated in red. NLS, nuclear localization sequence; CTBP, C-terminal-binding protein; AA, amino acid

As previously reported, the *MECOM* gene contains 16 exons and encodes a 1051 amino-acid protein which is a zinc finger transcription factor called EVI1, a transcription factor involved in embryogenesis, homeostasis of the hematopoietic stem cell compartment and megakaryocyte differentiation [3] (Fig. 3). Missense pathogenetic variants of EVI1 may reduce its interaction with DNA and/or other transcription factors [25]. Another report suggested *MECOM* being a candidate gene for hereditary hematological malignancies [26]. The novel variant (c.2285G > A, p.Arg762Lys) detected in our patient’s buccal swab and peripheral blood, and the same mutation was not detected in the parents who were asymptomatic and with normal blood counts. The results strongly suggest that the variant was a germ line mutation and disease-causing mutation. The novel *de novo* variant of *MECOM* gene is located in exon 11. Another research reported a splicing variant (NM_001105078:c.2285 + 1G > A) which mutated at the same site. So we speculated that both of the variants induced transcriptional dysregulation, similar to that caused by missense variants at the 8^th^ or 9^th^ ZF motif according to the functional analysis [17]. To clarify whether haploinsufficiency or a dominant-negative effect of the novel variant may be a causative mechanism of RUSAT-2 will require further functional studies (Fig. 3). More cases of RUSAT-2 from different ethnic populations are required for this to be validated.

Definitive treatment is a hematopoetic stem cell transplantation. Of the 38 reported patients with *MECOM*-associated syndrome transplanted, 34 engrafted while four died of transplant-related complications [1–4, 7, 9–23].

In conclusion, we reported a novel *de novo* missense mutation in (c.2285G > A, p.Arg762Lys) in the *MECOM* gene in a Chinese boy with RUSAT-2. This finding not only contributed to better genetic counseling, but also expanded the pathogenic mutation spectrum of *MECOM* gene. We also highlight the genetic analysis in all IBMFS and HSCT is the definitive treatment. Further studies are required to elucidate the molecular pathology underlying the development of RUSAT.

### Electronic supplementary material

Below is the link to the electronic supplementary material.

Supplementary Material 1

## Data Availability

The original contributions presented in the study are included in the article/supplementary materials. Further inquiries can be directed to the corresponding authors.
