# Supplementary material for: A novel missense mutation in the MECOM gene in a Chinese boy with radioulnar synostosis with amegakaryocytic thrombocytopenia
Source: BMC Pediatr. 2024 Jan 20;24:62. doi: 10.1186/s12887-024-04552-1 (PMC10799460; doi:10.1186/s12887-024-04552-1)
Supplement: Supplementary file 1 — Supplementary Material 1 [file 12887_2024_4552_MOESM1_ESM.docx]

**Supplemental Table 1 Clinical characteristics of the** **64 patients with *MECOM*-associated syndrome**

| **Charateristics** | **Value, no. (%)** |
| --- | --- |
| **Age at HSCT, y**  ≤0.5  >0.5 and <3  ≥3  unkonwn | 10 (15.6)  17 (26.6)  7 (10.9)  4 (6.2) |
| **Family history**  Simplex  Familial | 50 (78.1)  14 (21.9) |
| **Hematological characteristics**  Pancytopenia  Thrombocytopenia  Anemia  No cytopenia | 36 (56.2)  16 (25.0)  8 (12.5)  8 (12.5) |
| **Physical abnormalities**  RUS  Other skeletal malformation  Cardiac disorder  Kidney disorder  Hearing imparied  Neurological disorder  Skin, nail, or facial abnormalities  Precocious puberty | 29 (45.3)  27 (42.2)  17 (26.6)  6 (9.4)  9 (14.1)  11 (17.2)  15 (23.4)  4 (6.3) |
| **Immune dysfunction** | 10 (15.6) |
| **Myeloid malignancy** | 4 (6.3) |
| **HSCT** | 38 (59.4) |
| **Mutation types**  missense  nonsense  splice  deletion  frameshift  unknown | 35 (54.7)  6 (9.4)  5 (7.8)  8 (12.5)  6 (9.4)  4 (6.3) |
